# Serial versus parallel processing in mid-level vision: filling-in the details of spatial interpolation

**DOI:** 10.1093/nc/niv007

**Published:** 2015-10-02

**Authors:** Michele A. Cox, Alexander Maier

**Affiliations:** Department of Psychology, College of Arts and Science, Vanderbilt University, Wilson Hall, 111 21st Avenue South, Nashville, TN 37240, USA

**Keywords:** perceptual organization, mid-level vision, contour completion, filling-in, spatial interpolation

## Abstract

The relationship between boundary completion and surface filling-in, two core mechanisms of mid-level vision, remains unclear. Here, we integrate recent empirical findings to shine new light onto the neural mechanisms of boundary completion and surface filling-in as well as their relation to each other. Specifically, we discuss several psychophysical and neurophysiological studies that, when taken together, support a model where object boundaries and visual surfaces are interpolated in parallel, with one process impacting the other. We suggest that visual boundary completion and surface filling-in are two interacting processes that are supported by neural processes that are distributed throughout several areas of the early visual system.

## Serial and parallel processing for object vision

**Figure 1:**
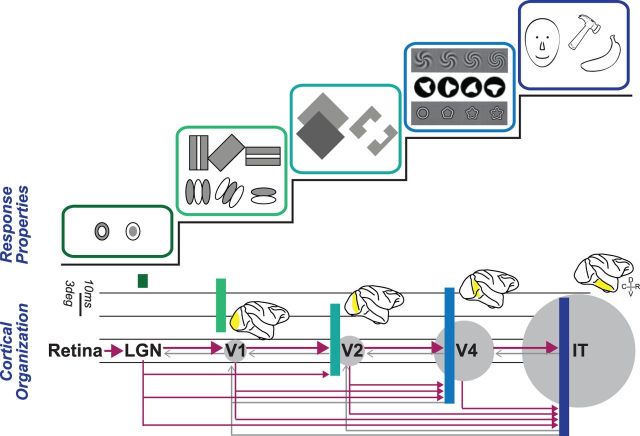
Simplified (and incomplete) scheme of the hierarchical organization of the primate visual system. Neurons in the lateral geniculate nucleus (LGN) and V1 respond to highly localized patches of light or darkness, irrespective of the nature of the image and the objects within it. Neurons in adjacent cortical visual areas, such as V2 and V4, exhibit increasingly complex response properties. Neurons at the latest stages selectively respond to 3D objects and faces. Anatomical connectivity (arrows; magenta = feedforward, gray = feedback), neuronal response latencies (horizontal bars), and relative receptive field sizes (gray circles) all support the view of a series of processing steps based on increasing neuronal convergence. Icons reprinted from Gallant JL, Shoup RE, Mazer JA. A human extrastriate area functionally homologous to macaque V4. *Neuron* 2000;**27**: 227–35, with permission from Elsevier; and Pasupathy A, Connor CE. Population coding of shape in area V4. *Nat Neurosci* 2002;**5**: 1332–38, with permission from Macmillan Publishers Ltd.

Visual perception is thought to arise from a series of hierarchically organized processing steps that extract increasingly complex visual features from raw sensory input (Felleman and Van Essen, 1991). This idea of increased convergence across a linear sequence of discrete visual processing steps is consistent with both the pattern of anatomical connections between visual brain areas (Fig. 1, bottom) as well as the longer onset times of responses to visual stimuli and the increasingly complex response preferences of neurons at later stages of processing (Fig. 1, top). However, this hierarchical scheme of visual processing also contains elements of parallel processing that are mediated via feedback connections that modulate neuronal responses at earlier stages of processing (Bullier and Nowak, 1995; Hochstein and Ahissar, 2002; Lamme, Supèr and Spekreijse, 1998; Lee *et al*., 1998; Zeki and Shipp, 1988).

**Figure 2:**
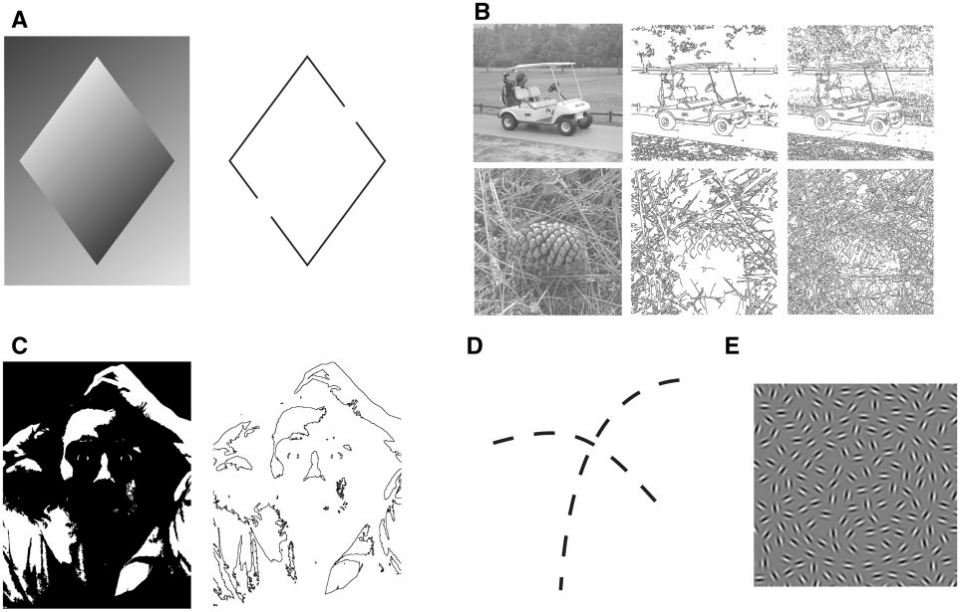
Edge detection and boundary completion. (a) Some parts of the diamond are lighter than the background and other parts are darker than the background. As the dark to light (or light to dark) gradient is continuous, two points along the diamond’s edge match the luminance of the background. These points are indicated by the gaps in the line drawing to the right. Despite the absence of a discrete luminance edge at these locations, we perceive a continuous boundary around the arrow. This perceptual effect demonstrates that the visual system can perceptually complete discontinuous edges by assuming that they are part of the same boundary (boundary completion; image adapted from Wolfe J, Kluender K, Levi D *et al*. (2011). *Sensation and Perception*. Macmillan). (b) Grayscale photographs of a golf cart and a pinecone with the output of two popular edge-detection algorithms next to them (middle, Canny; right, Rothwel). Notice how each algorithm extracts edges in different parts of the image, and how many of these edges are disjointed to the point of complete obfuscation (Heath *et al*., 1997). (c) This two-tone image (left) becomes nearly unrecognizable when only luminance edges are shown (right; original image *Le Désespéré* by Gustave Courbet). (d) Discrete visual edges are perceptually grouped into two coherent contours even in the absence of clear surface boundaries. (e) Example of a stimulus where a subset of grating patches is aligned to create a circular micropattern. Stimuli such as these are used to study perceptual contour linking and boundary completion (reproduced from Mundhenk TN, Itti L. Computational modeling and exploration of contour integration for visual saliency. *Biol Cybern* 2005;93: 188–212, with kind permission from Springer Science and Business Media).

At the main entry point of retinal information, the primary visual cortex (V1), neurons are highly selective for steep local luminance gradients, which aid the visual system in detecting the location and orientation of high contrast luminance edges within the two-dimensional (2D) retinal image (Hubel and Wiesel, 1977). However, without any additional processing steps, edge detection as a means to infer object boundaries falls prey to occlusion, low frequency luminance gradients, and other local and global image properties that prevent a clear distinction of pronounced visual objects (Fig. 2a–c).

## Boundary completion—a prerequisite for surface perception?

One way the visual system seems to overcome the inherent limits of edge detection is to perceptually complete object boundaries even if some edges are physically absent (Tversky, Geisler and Perry, 2004; Wertheimer, 1923). This phenomenon, known as “boundary completion,” is a form of perceptual interpolation whereby disjointed line elements are linearly interpolated in early visual cortex to form a cohesive contour (Fig. 2d–e; Field, Hayes and Hess, 1993).

An intuitive notion that follows from the above is that the early visual system performs a series of computational steps to extract increasingly complex visual features from retinal input. First, linear edges are extracted from the raw retinal input. Following this step, individual edges are spatially interpolated into outlines that delineate 2D image elements from each other and the background. Once these borders have been completed, each location within the visual field can be assigned to be within or outside of contour-defined shapes, and each enclosed shape is assigned respective surface attributes (filling-in).

Supporting this serial model, several psychophysical studies have demonstrated that image boundaries profoundly influence the perception of visual surfaces. In particular, a number of visual illusions suggest that a boundary’s contrast and color spreads rapidly across visual space to “fill in” the intervening surface (de Weerd, Desimone and Ungerleider, 1998; Paradiso and Nakayama, 1991; Pinna, Brelstaff and Spillmann, 2001). This interplay between boundary completion and surface filling-in can be readily observed in the Craik–Cornsweet–O'Brien effect (Cornsweet, 1970; Craik, 1966; O'Brien, 1959), illusory shapes (Kanizsa, 1976), neon color spreading (Van Tuijl, 1975), and afterimages (Shimojo, Kamitani and Nishida, 2001).

A recently described visual illusion demonstrates the causal influence of visual boundaries over perceptual surface filling more directly. In contour adaptation (CA), prolonged inspection of a contrast-inversing outline suppresses the perceptual visibility of a monochrome version of the encompassed surface if presented subsequently at the same location of visual space (Anstis, 2013). Critically, under these circumstances, only the neuronal populations representing the edge of the shape, not the shape’s surface, are fatigued by adaptation. Nevertheless, CA renders the entire shape perceptually invisible for up to several seconds, as if no visual surface was presented (Cox *et al*., 2014).

A second recently described visual illusion that strikingly demonstrates the deterministic effect of boundaries on surface filling-in employs perceptual afterimages. Typically, when a colored shape is viewed for a prolonged period of time and then removed, a shape with complementary color (i.e., an afterimage) is briefly perceived at the same location in visual space. However, the perceptual outcome of such afterimages can be profoundly altered by imposing a divergent contour (van Lier, Vergeer and Anstis, 2009). More specifically, adaptation on the same two-tone image can produce multiple, differently colored afterimages depending on the shape of the contour presented right after the colored stimulus. Both this afterimage illusion and CA seem to be parsimoniously explained by the assumption of visual surface filling-in following, and indeed being determined by, surrounding boundaries.

Several neurophysiological studies suggest a similar rank order of boundary representations and surface perception. Voltage sensitive dye imaging (VSD) in monkey V1, for example, revealed that population responses initially correspond to individual stimulus elements (Gilad, Meirovithz and Slovin, 2013). Once this initial response has tapered, population responses remain enhanced for contour elements but are depressed for background elements of the image. In the same vein, intracranial recordings of laminar responses in monkey V1 (Self *et al*., 2013) demonstrated three distinct phases of activity. The initial volley of activity in the feedforward input layers of V1 occurs regardless of a figure’s precise location relative to the neuronal receptive fields under study. Following this transient indiscriminant response, recording sites with receptive fields that are spatially coincident with the figure boundary exhibit elevated activity in the feedback- recipient upper layers of V1. A third phase of activity restricted to the upper and lower layers of V1 is only observed when the receptive fields are coincident with the surface of the figure.

One interpretation of these findings is that neuronal processes related to visual boundary completion precede the computations underlying surface filling-in. This interpretation would be consistent with the causal effects of visual boundaries on surface perception discussed above. However, a strictly sequential view of visual spatial interpolation, where boundary completion serves as a precursor or prerequisite of surface filling, has been repeatedly challenged on theoretical grounds (Grossberg, 2003; Kogo and Wagemans, 2013; Neumann, Pessoa and Hansen, 2001). Generally, these models suggest that spatial interpolation arises from parallel, potentially interacting processes of boundary completion and surface filling-in. In light of these considerations, the neuronal effects observed in V1 might be better explained by the assumption that the signals underlying filling-in are due to feedback that requires more time to reach V1 than it takes for the local computations underlying boundary completion to conclude. Is there any further empirical evidence that supports such a parallelized model of spatial interpolation?

## Surface filling-in as a determinant of boundary completion

**Figure 3:**
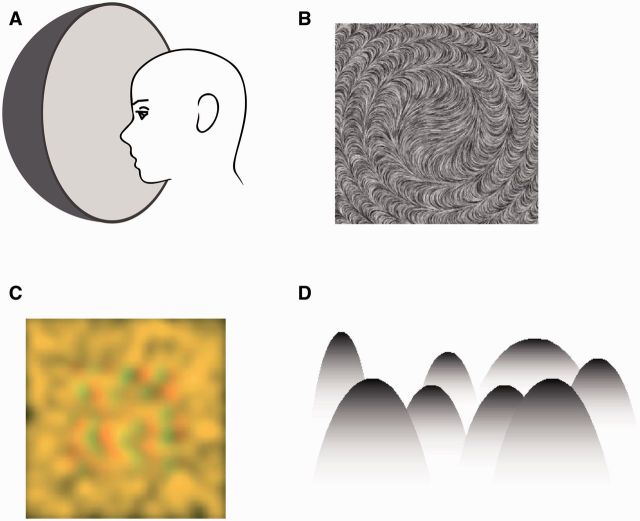
Boundary-independent visual surface perception. (a) Schematic of a Ganzfeld apparatus used to create the perception of a homogenous visual field consisting of a single surface. (b) A smoothly varying, orientation-defined texture gives the impression of a surface without clear or abrupt boundaries (reproduced from Ben-Shahar O. Visual saliency and texture segregation without feature gradient. *Proc Natl Acad Sci USA* 2006;**103**: 15704–09. © (2006) National Academy of Sciences, USA). (c) Random-dot stereogram with high spatial frequency component removed (courtesy of Blake R). When viewed through red-green glasses, a diffuse surface is perceived. (d) Mountain-shaped objects defined by a gradient. Where the gradient luminance matches the background, the sense of distinct visual boundary is lost (reproduced from Kitaoka A, Gyoba J, Sakurai K. The visual phantom illusion: a perceptual product of surface completion depending on brightness and contrast. *Prog Brain Res* 2006;**154**: 247–62. © (2006) with permission from Elsevier).

Support for the claim that boundary completion is not required for surface filling-in is derived from observations that demonstrate that the visual perception of surfaces can be established independently of the perception of encompassing boundaries (Fig. 3). One of these situations arises under viewing conditions that produce the impression of a surface without an explicit boundary. A simple example of such a boundless visual surface is the homogeneous image created with a Ganzfeld apparatus (Fig. 3a). Under these extraordinary circumstances, a visual surface is readily perceived without any delineating boundary (Gibson, 1950; Metzger, 1930). Another psychophysical observation which exemplifies that boundary completion and surface filling-in are carried by two separable, independent processes is that perceptually completed boundaries and surfaces add cumulatively to increase the saliency of visual objects (Machilsen and Wagemans, 2011). In the same vein, backward masking experiments using shapes and their component parts (i.e., lines and angles) show that test stimuli are strongly masked by completed shapes but only weakly masked by the individual line elements that constitute their boundaries (Lo and Zeki, 2014). Both these observations are in line with the assumption that surface filling may occur independently and in parallel to boundary completion.

**Figure 4:**
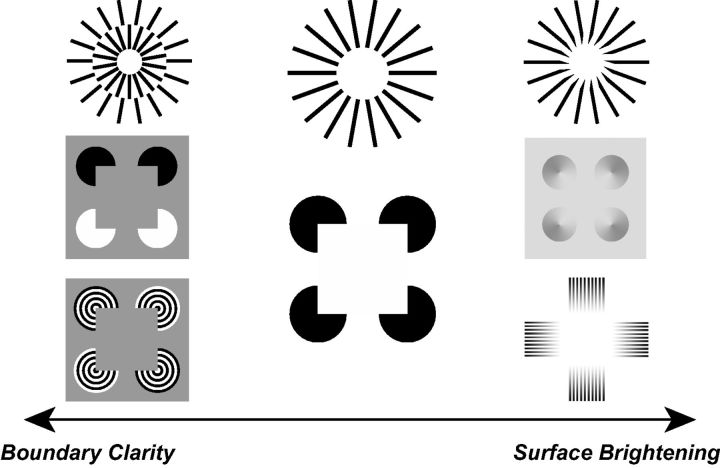
Modifications of the classic Ehrenstein illusion, Kanizsa figure, and Mach Band effect that demonstrate how certain stimulus conditions can disassociate boundary clarity from surface brightness. Images: Top row inspired by the work of Kennedy J and Parks TE; left middle and bottom inspired by Grossberg (2003); middle left from (reproduced from Kitaoka A, Gyoba J, Sakurai K. The visual phantom illusion: a perceptual product of surface completion depending on brightness and contrast. *Prog Brain Res* 2006;**154**: 247–62. © (2006) with permission from Elsevier); bottom left courtesy of Stubbs A.

Another line of evidence pointing toward the existence of surface filling mechanisms that are dissociable and partially independent from boundary completion stems from the phenomenology of illusory figures (also known as subjective contours). Notably, for certain classes of illusory figures, such as the popular Kanizsa square (Kanizsa, 1976), the central region of the perceptually evoked figure is generally perceived as brighter than the surrounding background despite the fact that these areas are physically identical and only separated by a subjective borderline (Fig. 4, center). What is more, there are stimulus variants that demonstrate this illusory brightness without eliciting a well-defined subjective boundary, an effect that is somewhat reminiscent of neon color spreading (Fig. 4, right). Yet, different versions of the stimulus evoke a vivid subjective contour without the concurring change in perceived brightness (Fig. 4, left). In other words, the phenomenology of certain illusory figures suggests that boundary completion and the spatial spread of a visual property such as brightness or color across a surface are dissociable and do not depend upon each other.

Since the perception of illusory figures requires interpolation of both boundaries and surfaces across different regions of a physically homogeneous visual field, this class of stimuli also provides a unique opportunity for studying the neurophysiological correlates and the respective spatiotemporal relationship between boundary completion and surface filling-in. Two recent studies using these stimuli have posed challenges to a strictly serial hierarchical model of boundary completion and surface filling-in. In the first study, transcranial magnetic stimulation (TMS) was used to investigate the role of both early and late cortical areas in the visual processing of illusory figures. Online TMS was used to disrupt signaling in V1/V2 and in the shape-selective lateral occipital area (LOC) at various time points while participants performed a discrimination task involving a Kanizsa-type illusory figure (Wokke *et al*., 2013). Results suggest that both V1/V2 and LOC are causally involved in the perceptual completion of illusory figures. However, the critical time window during which focal TMS disrupted perception occurred earlier for LOC (∼100 ms) than for V1/V2 (∼160 ms). The temporal specificity with which TMS to V1 affected perception can be explained by a critical processing period during which surface (i.e., shape) specific feedback interacts with the edge extraction and boundary completion computed in V1/V2. In other words, the neuronal signals related to surface filling-in might interact with and modulate the neuronal signals associated with boundary completion.

**Figure 5:**
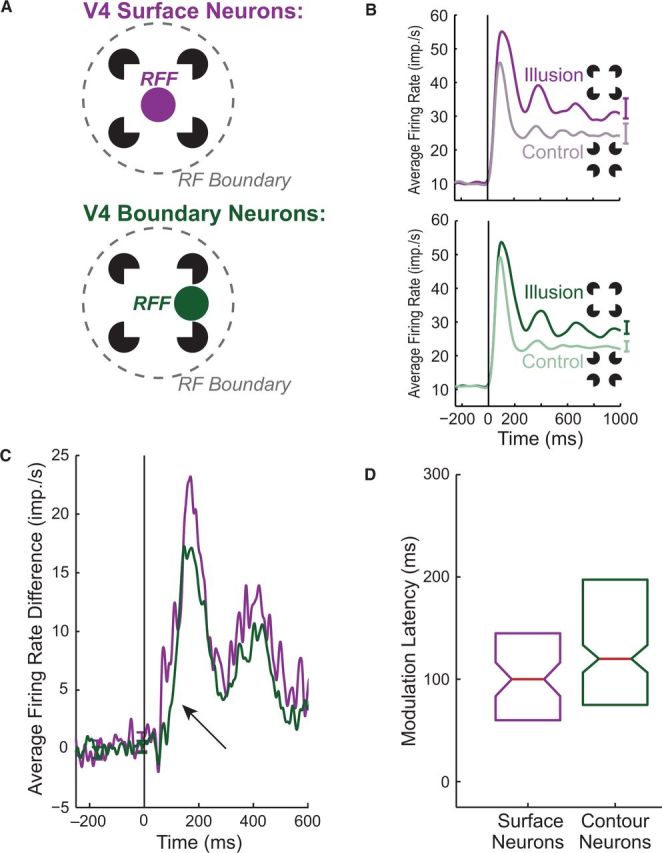
Single neuron data suggesting that the neuronal underpinnings of boundary completion and surface filling are dissociable in space and time. (a) Neurons recorded from macaque V4 defined as either “surface neurons” (purple) or “boundary neurons” (green) depending on the location of their peak retinotopic sensitivity (RFF) relative to the features of an illusory figure. (b) Average responses to the illusory figure (dark) and a rotated control stimulus (light) are shown for both populations. (c) Firing rate difference between conditions for both populations (black arrow highlights latency difference). (d) Relative response latency of illusory surface-related signals, defined as the time when the neuronal response evoked by the illusion and the control stimulus diverged, is indicated by the box plot. Note that the neuronal response modulation to the illusory figure emerges in surface neurons concurrent with, or even before, the signal in the contour neuron population. Reproduced from Cox MA, Schmid MC, Peters AJ *et al*. Receptive field focus of visual area V4 neurons determines responses to illusory surfaces. *Proc Natl Acad Sci USA* 2013;**110**: 17095–100, with permission from the National Academy of Sciences, USA.

Further support for non-sequential ordering of boundary completion and surface filling-in stems from recent single neuron recordings in macaque monkeys viewing Kanizsa-type illusory figures (Cox *et al*., 2013). In this study, spiking activity in area V4 was differentially affected by exposure to the illusory boundary, the illusory surface, or the physical edge. Specifically, V4 neurons showed stronger spiking responses for the illusion-promoting stimulus configurations compared to controls when their peak visual field sensitivity, or receptive field focus (RFF), was centered on the illusory surface or its encompassing subjective contour compared to being centered on the illusion-inducing image elements (Fig. 5a–b). Strictly, sequential models of visual spatial interpolation predict that neuronal responses to visual boundaries precede those for surfaces, which should extend to this illusion. However, the response enhancement for the illusory shape emerged in the population of surface-focused neurons at the same time, if not earlier, than in the contour-focused neurons (Fig. 5c–d). Taken together, these results suggest an active role of V4 neurons in boundary completion and surface filling-in, with the neural underpinnings of surface filling-in occurring simultaneous to—or perhaps even before—the process of boundary completion.

## Boundary completion and surface filling-in as parallel and interacting processes

The combined results of the empirical studies outlined above suggest that a certain degree of surface segregation, based on partial boundaries or other aspects of global stimulus configuration, may sometimes occur before surface boundaries are completely delineated by the visual system. Several possible relationships between surface filling-in and boundary completion can be conceptualized to explain these findings. One possibility is that both processes are initiated in parallel. While boundary completion operates on input from edge detection, the extraction of visual surfaces might operate on the low spatial frequency components of the retinal image that provide information about global variations in brightness and hue (Haynes, Lotto and Rees, 2004; Komatsu, Murakami and Kinoshita, 1996). Another possibility, though not completely exclusive from the former, is that the processes of surface extraction and boundary completion are initially separated but interact at later stages to compute visual shapes. Notably, this model allows for processes related to surface filling-in to solidify boundary representations, especially when boundaries are difficult to resolve or visually ambiguous. Neurons that signal border-ownership, that is, neurons that encode both the boundary as well as the surface that the boundary belongs to, might play a crucial role in this process. These neurons have been found throughout early visual cortex (V1-V4) (Zhou, Friedman and von der Heydt, 2000). Boundary completion and surface filling-in thus might be conceptualized as two distinct processing phases that either exist on the same horizontal plane within a larger visual hierarchy, or as two elements within an organizational scheme with a more flexible rank order.
